# Administration of aspirin tablets using a novel gel-based swallowing aid: an open-label randomised controlled cross-over trial

**DOI:** 10.1136/bmjinnov-2018-000293

**Published:** 2019-07-04

**Authors:** David John Wright, John F Potter, Allan Clark, Annie Blyth, Vivienne Maskrey, Giovanna Mencarelli, Sarah O Wicks, Duncan Q M Craig

**Affiliations:** 1 Pharmacy, University of East Anglia, Norwich, UK; 2 Medicine, University of East Anglia, Norwich, UK; 3 University of Bath, Bath, UK; 4 Department of Pharmacy, Health and Well-Being, University of Sunderland, Sunderland, UK; 5 School of Pharmacy, University College London, London, UK

**Keywords:** dysphagia, swallowing aid, bioequivalence, aspirin, salicylate levels, platelet function

## Abstract

**Introduction:**

To ease administration of medicines to people with dysphagia we developed and patented a gel formulation within which whole tablets could be inserted. The aim was to determine whether the gel would affect bioequivalence of uncoated aspirin tablet.

**Method:**

A gel containing gelatin, hydroxypropylmethylcellulose, citric acid, potassium sorbate and water was developed to maintain structure on tablet insertion and increase saliva production to lubricate the swallow.

In an open-label cross-over trial 12 healthy male volunteers were administered a 300 mg uncoated aspirin tablet with and without gel with a 7-day washout period. Blood salicylate levels, platelet activity and patient satisfaction were measured over 2 hours. Analysis was based on a random effects cross-over model.

**Results:**

The estimated mean ratio (90% CI) of effect on salicylate levels when comparing administration with and without gel was 0.77 (90% CI 0.40 to 1.47) for amount absorbed and 0.76 (90% CI 0.44 to 1.31) and on total ASP-arachidonic acid platelet activity 1.16 (90% CI 0.88 to 1.53) and maximum ASP-arachidonic platelet activity 0.98 (90% CI 0.79 to 1.22). These results are outside of the range allowable for the assumption of bioequivalence. Participants rated the taste of aspirin tablets significantly better when encapsulated in the gel (p<0.05).

**Discussion:**

We cannot assume that uncoated aspirin administration with and without gel is bioequivalent. Administration with gel resulted in reduced salicylate levels and therefore increased platelet function. Further research is required to determine the exact reason for this result. The results bring into question current processes for providing marketing authorisation for medical devices which are designed to aid swallowing.

## Introduction

Administration of tablets and capsules in people with dysphagia has been shown to be related to a threefold increase in medication error rate and this is largely attributed to the frequent need to modify the solid dose formulation by crushing or dispersing in water prior to administration.^1^ Guidance frequently recommends solid dose formulation alteration prior to administration in order to minimise the risk of choking and make oral bolus formation easier.^2^ Crushing modified release solid dose formulations can result in immediate dose release and therefore increase likelihood of patient harm as they receive the whole dose more rapidly than anticipated.^3^ Similarly, disrupting enteric coatings can increase likelihood of harm to the patient’s stomach, harm to the chemical entity or the drug being released higher up the gastrointestinal (GI) tract than anticipated.^3^ Consequently, in such cases, alternative routes of administration or formulations are required.^4^

Liquid formulations are available for most medicines and their prescription immediately overcomes concerns regarding bolus formation in those with oral phase dysphagia. The texture of liquid medicines for patients with pharyngeal phase dysphagia can however be inappropriate as they may increase the likelihood of aspiration.^3^ Due to the small market share and increased costs of production and storage, liquid medicines are more expensive than solid dose formulations and consequently cost is an additional barrier to their prescription.^5^ It is not possible for the liquid formulation to provide enteric coatings or sustained release characteristics, which are routinely seen with solid dose oral formulation, and consequently liquid medicines can require more frequent dosing and cannot be formulated to protect the stomach, active ingredients, or to ensure that these are released past the stomach and duodenum.

With a desire to provide patients with tablets or capsules intact but lubricated to ease the swallow and give the mouthfeel of a liquid medicine, we developed a new formulation gel which enabled tablets or capsules to be encapsulated prior to swallow, thereby removing the need for altering tablet form prior to administration. Using pharmaceutically inert materials, the gel (SMART Swallowing Aid) was designed and patented (US Patent Application No 12/866715, Japanese Patent Application No 2010-545564, European Patent Application No 09708982.5, Chinese Patent Application No 200980104351.4) to not react with the medicines or their coatings and to additionally become more lubricated when in contact with saliva. The formulation did not contain any sugar making it suitable for those patients on a controlled diet. The SMART Swallowing Aid prototype gel was developed with the proposed advantages of:

- Increasing the comfort of the swallow.
- Providing lubrication to the tablet thereby negating the need for concomitant administration of water.
- Eliminating the need to crush medication.
- Enabling medicines designed to be absorbed further down the GI tract to be delivered intact.
- Masking the taste of the medication.
- Protecting the GI mucosa, in particular the oesophagus, to reduce oesophagitis.^6^
- Increasing the weight of the tablet and thereby aid passage down the oesophagus into the stomach.
- Minimising accusations of administering medicines covertly^7^ by ensuring that the gel was transparent.

Other tablet swallowing aids are currently available (Pill glide,^8^ Medcoat^9^) which are designed either to coat the tongue or the tablet, thereby masking the flavour and making it easier to swallow the medication. All products consist of ingredients, which are considered to be safe for humans, and are believed not to affect absorption of the drug from the tablet. Using a standard in vitro model, Medcoat has been shown to not adversely affect tablet disintegration.^9^ While the products claim to not affect drug absorption, none have been tested in patients to determine bioequivalence. With concerns regarding patient safety and effectiveness surrounding the encapsulation of a tablet prior to swallowing and a desire to register SMART Swallowing Aid as a medicinal device we decided to undertake in vivo testing.

The aim of this study was to investigate the safety, efficacy and acceptability of SMART Swallowing Aid prototype. The primary objectives of this study were to establish bioequivalence of aspirin with and without SMART Swallowing Aid (drug plasma concentration and drug effect on platelet activity) and test the safety of SMART Swallowing Aid in human subjects. As a secondary outcome, the study determined whether the gel was easy and comfortable to swallow in comparison with the tablet administered with water and whether it separated from the tablet on swallowing.

## Method

The study consisted of a phase IV open-label randomised controlled cross-over trial, comparing the bioavailability of aspirin encapsulated within SMART Swallowing Aid with the bioavailability of aspirin alone administered with water.

### SMART Swallowing Aid

Gelatin, from a bovine origin and certified to be bovine spongiform encephalopathy/transmissible spongiform encephalopathy free, formed the main ingredient as it is odourless and tasteless and forms a gel at room temperature and melts around body temperature (<35^○^C). To improve the gel’s swelling and lubrication properties hydroxypropylmethylcellulose was added as it becomes less tacky on exposure to water and therefore in the presence of saliva will lubricate the formulation. Citric acid was added to increase saliva production and as a preservative. Potassium sorbate was also included as the amount of citric acid was insufficient alone to prevent microbial contamination. All ingredients were selected due to their wide routine use within pharmaceutical and food industries. The aspirin and gel combination was compared with aspirin alone in vitro via a model gut^10^ to confirm bioequivalence.

### Trial

**Figure 1:**
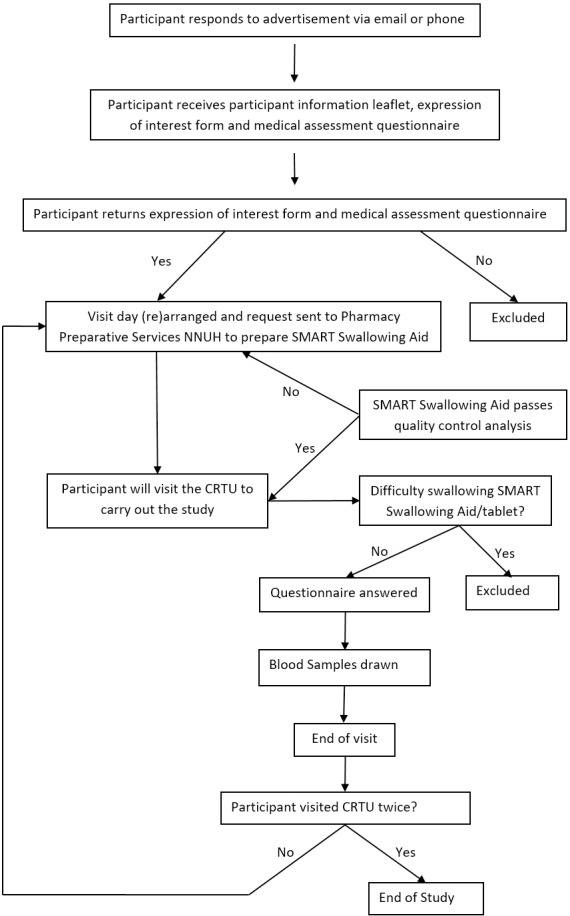
Study progress flow chart. CRTU, Clinical Research and Trials Unit; NNUH, Norfolk and Norwich University Hospital.

The overall trial process is summarised in figure 1. Twelve healthy male volunteers were recruited via poster advertising within the University of East Anglia (UEA) and the Norfolk and Norwich University Hospital. As we expected small variation within individuals and we have evidence of equivalence based on lab-based approaches we felt that a sample size of 12 individuals would be sufficient to have high power to demonstrate bioequivalence.

The study *inclusion criteria* were participants who were:

1. Healthy.
2. Male.
3. Aged 18–35 years.

We selected only male participants as Aspirin should be used with caution in pregnancy and then at low dose. Testing in one gender also limits pharmacokinetic variation.

The study *exclusion criteria* were:

1. Student at either the UEA School of Pharmacy or Medicine.
2. Allergy or hypersensitivity to aspirin (or other non-steroidal anti-inflammatory drugs) or formulation ingredients.
3. Lactose intolerance or coeliac disease.
4. Dysphagia or difficulty swallowing.
5. Active peptic ulceration.
6. Haemophilia or other bleeding disorders.
7. Taking aspirin regularly or who have taken it in the last 7 days.
8. Taking other medications, particularly those containing salicylates.
9. Parallel participation in another research study.
10. Related to or living with any member of the study team.
11. Language difficulties.

Participants were withdrawn if they suffered an adverse event or reaction in response to aspirin or the swallowing aid or if they demonstrated difficulty in swallowing using SMART. The study was planned to be terminated if the gel was deemed a choking hazard.

Participants were randomised using sealed envelopes, which were presented in numerical order to each participant at the start of the first visit day. Block randomisation was used with a block size of 4. While this study could not be blinded, only the study statistician was aware of the randomisation details prior to the visit days.

Participants attended the Clinical Research and Trials Unit (CRTU) based at the Norfolk and Norwich University Hospital having not consumed any food or drink for at least 2 hours prior to each visit.

**Figure 2:**
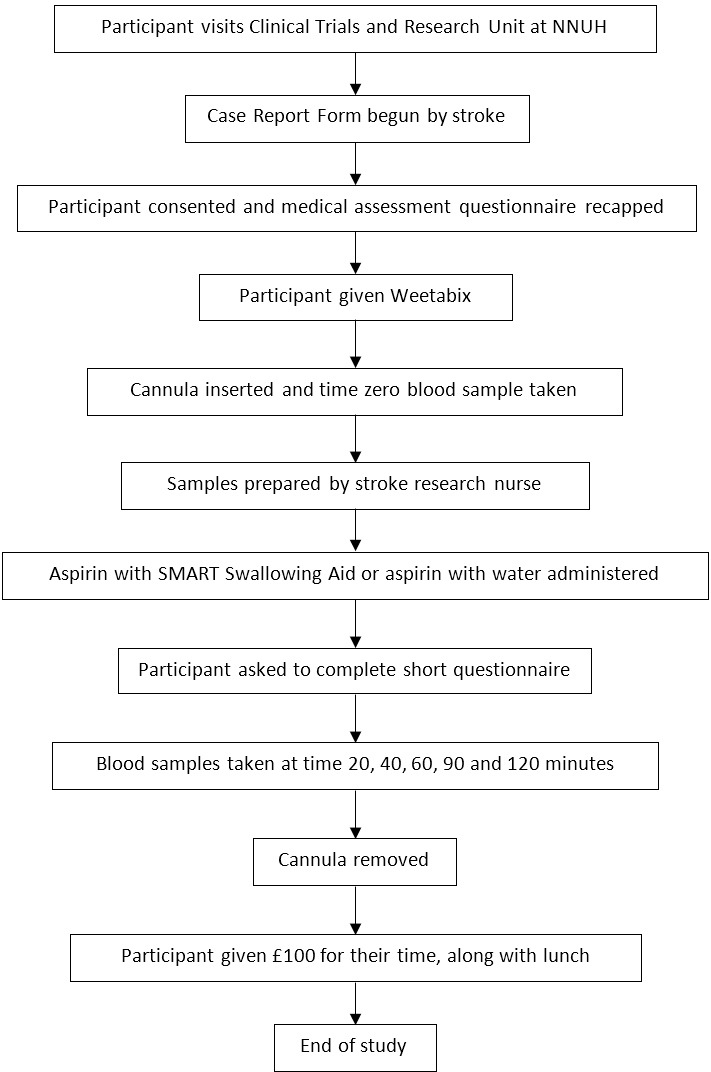
Tablet encapsulated within SMART Swallowing Aid. NNUH, Norfolk and Norwich University Hospital.

Dependent on randomised allocation each participant was asked to swallow either one SMART Swallowing Aid containing one 300 mg aspirin uncoated tablet (figure 2), or to swallow one 300 mg aspirin uncoated tablet with a glass of potable water.

Aspirin should be taken with food to avoid the risk of stomach ulceration, and consequently to standardise stomach contents prior to administration all participants were given two Weetabix on arrival with a measured volume of semiskimmed milk. Aspirin is known to exhibit a half-life of 15–20 min, with that of its metabolite salicylate being 3–6 hours. A washout period of 1 week allowed elimination of aspirin and its metabolite in the region of 504 and 28 half-lives, respectively, which was adequate for the purposes of this study. Participants were asked not to take aspirin or any medications containing aspirin or salicylates for 7 days prior to each data collection event and this was confirmed on arrival at the visit day.

Venous blood samples were taken at time 0 and 20, 40, 60, 90 and 120 min after the dose.^11^ Serum salicylate levels were measured with Advia 2400, Clinical Chemistry System by contract research company Viapath, Kings College, London.^12^ Platelet function was measured by impedance aggregometry using the Multiplate system (Verum Diagnostica, Munich, Germany).^13^ Platelet function included both measurements of effect on arachidonic acid and collagen.

Participants completed a questionnaire after taking the tablet with SMART Swallowing Aid and after taking the tablet with water. Questionnaires consisted of three visual analogue scales (VAS) in response to ‘The tablet was easy to swallow’, ‘The tablet was comfortable to swallow’ and ‘I could taste the tablet during the swallow’. The lower the score the more positive the response. Additionally, a single yes/no question ‘The gel aid broke/the tablet separated from the gel aid’ was included when the tablet was administered with the gel.

**Figure 3:**
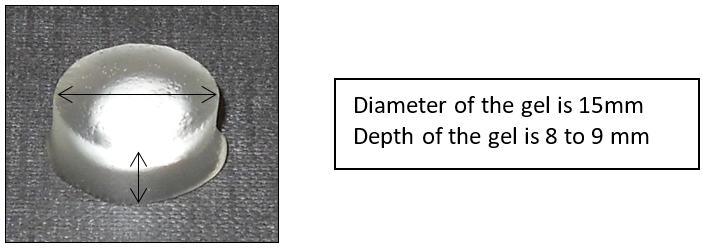
Visit progress flow chart.

Participants then returned after at least 1 week to repeat the study with the alternative method of administration and were remunerated for their participation. The process within the CRTU is summarised in figure 3.

### Outcomes

The outcome measures were: (1) the maximum concentration of salicylate; (2) area under the curve (AUC) of the concentration of salicylate (salicylate concentration measured at 0, 20, 40, 60, 90 and 120 min after swallowing); (3) the maximum collagen; (4) the AUC of the collagen; (5) the maximum ASP-arachidonic acid AUC; and (6) the AUC of ASP-arachidonic acid. Collagen AUC and ASP-arachidonic acid AUC are measured at 0, 20, 60 and 120 min after swallowing.

### Data analysis

Analysis was undertaken using the software package SAS 9.4.

Data generated from the participant taking SMART Swallowing Aid will be referred to as T (or test) and the data generated from the participant taking the non-gel as R (or reference).

### Derived variables

The AUC for aspirin serum concentration was calculated using the trapezoidal rule using the equation:

$$\begin{array}{ll} AUC= & \frac{\left(C_{0}+C_{20}\right)}{2}\times 20+\frac{\left(C_{20}+C_{40}\right)}{2}\times 20+\frac{\left(C_{40}+C_{60}\right)}{2}\times 20\\ & +\frac{\left(C_{60}+C_{90}\right)}{2}\times 30+\frac{\left(C_{90}+C_{120}\right)}{2}\times 30 \end{array}$$

where Ct is the aspirin serum concentration at time t. Any missing or incomplete data were replaced using last observation carried forward in order to calculate the AUC.

AUC for the platelet function test was calculated in a similar fashion.

Cmax for both aspirin serum concentration and platelet function test was calculated as the maximum value of the variables at the observed time points.

As an equivalence study, only individuals who adhered to the protocol were included in the analysis.

### Average bioequivalence

In order to demonstrate average bioequivalence (ABE), both the mean AUC and the mean Cmax for treatment should not significantly differ from the mean AUC and mean Cmax for control using a two one-sided test procedure. The trial team set the limits that ABE would be demonstrated if the 90% two-sided CI for the mean difference falls within the acceptance limits of –ln(1.25).^14^ To be precise, if µT denotes the true mean log(AUC) {or log(Cmax)} for those with the test and µR for those with the reference group, ABE is demonstrated if there is good evidence that:

$$0.8\leq exp\left(\mu_{T}-\mu_{R}\right)\leq 1.25$$

The model used to estimate ABE was based on a random effects cross-over model using the Kenward-Roger df.^14^

### Population bioequivalence

The level of both population bioequivalence (PBE) was measured using the standard guidelines issued from the Food and Drug Administration and estimated using a mixed model approach to estimate the within-subject variances required for the measured PBE. For PBE, the metric is given by equation whose 90% CI must be below zero.^14^

For all ABE and PBE and random effects (participants as random), a cross-over model was used to estimate the required parameters. The aggregate measures are functions of the estimated variances and easily calculable from the computer output.

It is not possible to estimate the power of this study since there are not enough data on which to base an estimate of the within-subject SD. Although given that SMART Swallowing Aid is unlikely to have a large impact on the AUC or Cmax, the within-subject SD was likely to be small and hence the power was likely to be high. It should be noted, however, that cross-over trials used to assess for PBE generally use in excess of 20–30 subjects so these results may be more exploratory than confirmatory.

### Questionnaire analysis

The ease of swallowing and the comfort of swallowing and taste obtained from the questionnaires were analysed using a non-parametric Mann-Whitney test.

### Regulatory approvals

The trial was registered with EudraCT 2011-005208-14 and ISRCTN 13972867.

## Results

Twelve healthy male volunteers were randomised to receive either ‘Gel/no gel’ or ‘No gel/gel’. All participants who were randomised finished the trial and no participant was lost to follow-up. All participants took the intervention as intended and no adverse events were observed during the course of this trial.

The analysis was performed in compliance with the statistical analysis plan, due to the nature of bioequivalence it is necessary to know the treatment (T) and the reference (R) so this analysis was not blinded.

### Bioequivalence results

A summary of the outcome measure at the end of each period is given in table 1. This shows roughly equal means for collagen and ASP-arachidonic acid in each period and each sequence, but a slight difference in salicylate AUC and Cmax.

**Table 1:** Summary statistics of outcome measures

	Sequence			
	**No gel/with gel**		**With gel/no gel**	
	**Period 1**	**Period 2**	**Period 1**	**Period 2**
Log AUC collagen	8.98 (0.19)	9.04 (0.19)	8.98 (0.21)	8.86 (0.18)
Log Cmax collagen	4.43 (0.13)	4.42 (0.12)	4.37 (0.23)	4.24 (0.11)
Log AUC ASP-arachidonic acid	8.80 (0.42)	8.98 (0.33)	8.76 (0.54)	8.63 (0.24)
Log Cmax ASP-arachidonic acid	4.66 (0.22)	4.59 (0.17)	4.41 (0.52)	4.38 (0.18)
Log AUC salicylate	6.02 (1.07)	6.25 (0.83)	5.61 (1.06)	6.36 (0.66)
Log Cmax salicylate	2.24 (0.86)	2.40 (0.74)	1.83 (0.82)	2.55 (0.54)

ASP, aspirin; AUC, area under the curve.

**Figure 4:**
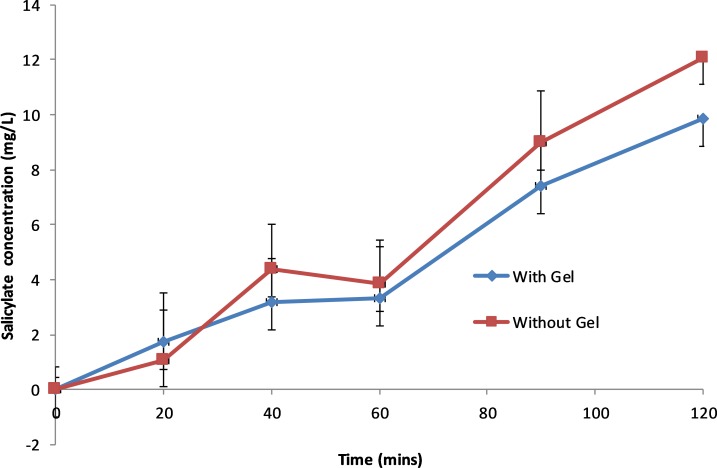
Salicylate concentration (mg/L) over time for uncoated aspirin administered with and without gel.

**Figure 5:**
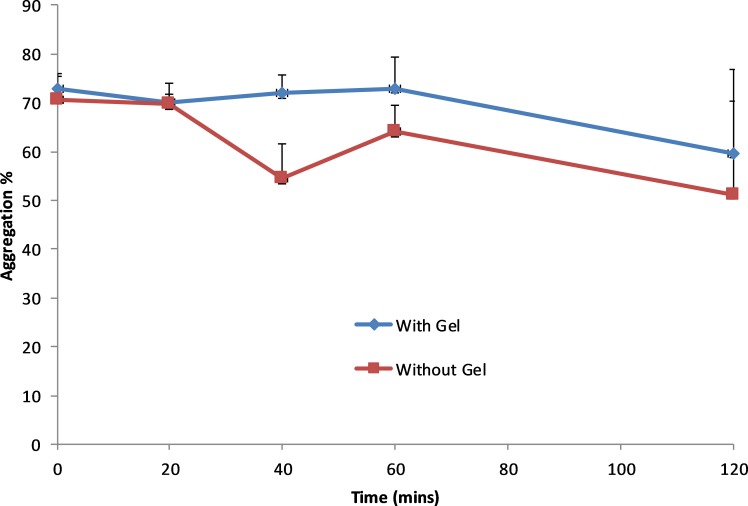
Collagen platelet activity over time for uncoated aspirin administered with and without gel.

**Figure 6:**
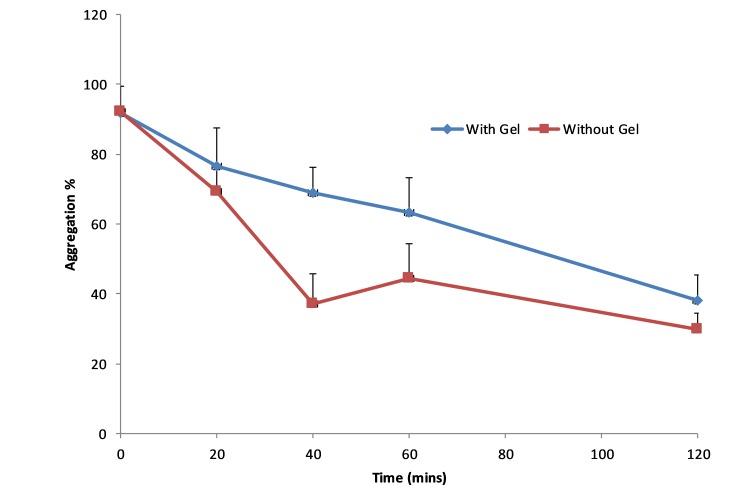
ASP-Arachidonic acid platelet activity over time for uncoated aspirin administered with and without gel.

ABE results are presented in table 2. These have been estimated based on random effects cross-over model using the Kenward-Roger df.^14^ These show that since the 90% CI does not fall within the (0.80, 1.25) window, ABE cannot be concluded. The change in salicylate concentrations, collagen and ASP-arachidonic acid activity over time is shown in figures 4–6, respectively. The figures show an increase in salicylate levels and corresponding reduction in platelet activity as measured by ASP-arachidonic acid, but limited or no change in platelet activity as measured by collagen. Giving the aspirin with the gel results in lower salicylate concentrations and higher platelet activity.

**Table 2:** Estimate of ratio of means (with gel/no gel) and 90% CI

Variable	Estimate of mean ratio	90% CI
Log AUC collagen	1.09	(0.96 to 1.24)
Log Cmax collagen	1.07	(0.99 to 1.16)
Log AUC ASP-arachidonic acid	1.16	(0.88 to 1.53)*
Log Cmax ASP-arachidonic acid	0.98	(0.79 to 1.22)*
Log AUC salicylate	0.77	(0.40 to 1.47)*
Log Cmax salicylate	0.76	(0.44 to 1.31)*

*Outside of range allowable to enable assumption of bioequivalence.

ASP, aspirin; AUC, area under the curve.

PBE was estimated using the metric provided in Jones and Kenward (2003, p 343).^14^ This metric is essentially a combination of the mean difference (squared) and the variances of the treatment response. The important factor is the upper 90% CI bound for the metric. If this is below zero then PBE can be declared. It should be noted that if ABE has not been demonstrated it is unlikely that PBE can be demonstrated. The values for each outcome are given in table 3. These show that T and R are PBE for collagen, but not for ASP-arachidonic acid or salicylate.

**Table 3:** Lower 90% CI bound for PBE metric

Variable	Lower bound
Log AUC collagen	−0.01
Log Cmax collagen	−0.02
Log AUC ASP-arachidonic acid	0.19*
Log Cmax ASP-arachidonic acid	0.16*
Log AUC salicylate	0.54*
Log Cmax salicylate	0.44

*Outside of range allowable to enable assumption of bioequivalence.

ASP, aspirin; AUC, area under the curve; PBE, population bioequivalence.

### Questionnaire results

The summary of the questionnaire VAS responses and formal comparisons is given in table 4. This shows that there is a significant treatment effect for taste with the gel group having a lower mean score than the no gel group. Out of the 12 individuals, 11 reported that the gel did not break and one reported that ‘first tablet spat out as it separated from gel, second tablet didn't separate.’

**Table 4:** Summary statistics of questionnaire data

	Sequence				P value	Carry-over P value
	No gel/with gel		With gel/no gel			
	Period 1	Period 2	Period 1	Period 2		
Ease of swallowing	9.55 (0.4)	8.73 (1.59)	7.48 (2.07)	9.05 (1.08)	0.1495	
Comfort	9.13 (1.50)	7.75 (1.97)	8.93 (0.77)	9.22 (1.23)	0.1087	
Taste	4.73 (4.57)	0.58 (0.62)	1.47 (1.54)	4.42 (4.35)	0.0370	0.5204

## Discussion

The study successfully recruited to target with no dropout. There were no safety issues identified during the trial. It cannot, however, be stated that bioequivalence was demonstrated when taking aspirin with and without the gel. The salicylate levels and amount absorbed seemed to be greater without the gel, which corresponded with lower platelet function test results. As anticipated, the patients who swallowed aspirin coated in the gel found the taste to be better. Furthermore, although water was not provided with the aspirin tablet when it was coated in the gel no significant effect on ease of swallow or swallow comfort was detected.

As a randomised controlled cross-over study we were able to use a relatively small sample size to detect reasonable differences in bioequivalence. Although the trial was open label and this was unavoidable due to the nature of what was being tested we would not expect this to adversely affect clinical biomarkers.

We chose to give Weetabix prior to administration of the tablet with and without gel as the trial occurred in the morning and this is a common breakfast within the UK. As a randomised control cross-over trial any effect that it may have itself on absorption will have been taken into account.

While we followed a previously reported process for undertaking an evaluation to assess salicylate levels after dose, the results show that we did not capture the full pharmacokinetic profile.^11^ Tmax for normal coated aspirin is 2 hours^15^ and therefore considering the results seen, either the Cmax or Tmax is unlikely to be the same for aspirin when given with or without gel.

The results suggest therefore that the gel may be reducing aspirin absorption, resulting in increased platelet activity. Our in vitro test for bioequivalence identified an average difference of 6 s in disintegration time and this we did not deem sufficient to significantly affect bioequivalence. The encapsulation may, however, be delaying the release of aspirin in vivo more than that identified in vitro and a longer follow-up time may have resulted in similar total aspirin release. An alternative hypothesis may be that the water content in the gel, which is in close contact with the uncoated tablet, may be hydrolysing the aspirin and therefore making less available for absorption and metabolism to salicylate in the first instance. Finally, an ingredient within the gel itself may be incompatible with aspirin and either physically or chemically interacting with the molecule to reduce absorption. The ingredients were selected due to their largely inert nature, however this may not be the case with this specific molecule.

While one option maybe to test the gel with different medicines which are known to be less susceptible to water or less likely to break down before the gel is absorbed, we would suggest that it would be more efficient to reformulate the gel to ensure that it is unlikely to affect absorption of all medicines.

The finding that reductions in aspirin levels were related to increased platelet function as measured by arachidonic acid is to be expected as this is seen as an appropriate measure for this purpose^16^ whereas platelet binding to a collagen surface has been shown to be less sensitive to changes in aspirin levels.^17^

It could be argued that we selected a chemical entity and formulation which was more likely to be affected by the encapsulation and therefore this may not have provided an ideal model. However, aspirin was selected because it is frequently prescribed in individuals with dysphagia and due to it being very likely to react with ingredients and therefore a positive result would have provided greater reassurance regarding the inert nature of the gel.

The results bring into question the fact that other similar products have been allowed to be marketed to be used to ease medication administration without in vivo testing. Recent research has shown that mixing medicines’ food thickeners significantly reduces their effectiveness^18 19^ and although the extent of interaction and contact with swallowing aids and medicines is less, it seems that the likelihood of interaction needs to be explored more extensively prior to award of marketing authorisation.

We therefore have to conclude that in using the gel to enable aspirin to be swallowed without either crushing or the addition of water we cannot assume bioequivalence and therefore further research is required to determine the exact reason for this result and to develop a product, which is less likely to affect bioequivalence.
